# Construction of a system using a deep learning algorithm to count cell numbers in nanoliter wells for viable single-cell experiments

**DOI:** 10.1038/s41598-017-17012-x

**Published:** 2017-12-04

**Authors:** Takashi Kamatani, Koichi Fukunaga, Kaede Miyata, Yoshitaka Shirasaki, Junji Tanaka, Rie Baba, Masako Matsusaka, Naoyuki Kamatani, Kazuyo Moro, Tomoko Betsuyaku, Sotaro Uemura

**Affiliations:** 1 Pulmonary Division, Department of Medicine, Keio University School of Medicine, 35 Shinanomachi Shinjuku-ku, Tokyo 160-8582, Japan; 2 Department of Biological Sciences, Graduate School of Science, The University of Tokyo, Bunkyo-ku, Tokyo, Japan; 3 RIKEN Center for Integrative Medical Sciences, Tsurumi-ku, Yokohama, Kanagawa, Japan; 4 StaGen Co. Ltd, Taito-ku, Tokyo, Japan

## Abstract

For single-cell experiments, it is important to accurately count the number of viable cells in a nanoliter well. We used a deep learning-based convolutional neural network (CNN) on a large amount of digital data obtained as microscopic images. The training set consisted of 103 019 samples, each representing a microscopic grayscale image. After extensive training, the CNN was able to classify the samples into four categories, i.e., 0, 1, 2, and more than 2 cells per well, with an accuracy of 98.3% when compared to determination by two trained technicians. By analyzing the samples for which judgments were discordant, we found that the judgment by technicians was relatively correct although cell counting was often difficult by the images of discordant samples. Based on the results, the system was further enhanced by introducing a new algorithm in which the highest outputs from CNN were used, increasing the accuracy to higher than 99%. Our system was able to classify the data even from wells with a different shape. No other tested machine learning algorithm showed a performance higher than that of our system. The presented CNN system is expected to be useful for various single-cell experiments, and for high-throughput and high-content screening.

## Introduction

Recently, it has been realized that model systems for cellular experiments provide an oversimplified view of the *in vivo* reality that does not take cellular heterogeneity into account. To investigate such cellular heterogeneity, single-cell studies have gained importance^1–4^. Characterization of individual cells may be possible using technologies such as single-cell RNA-seq.

Single-cell studies have demonstrated that individual characteristics of secreted cytokine profiles are present in single cells, even in macrophage populations^5^. For such studies, a large number of viable single cells have to be analyzed to understand the precise composition of the cellular population. To this end, cells are often distributed into many nanoliter well trays followed by cell counting. Cell counting is rather difficult because cells are neither stained nor fluorescence-labeled. Manual or visual inspection cell counting methods are the current gold standard, although they may have more pressing limitations in future large-scale applications with large numbers of samples.

Automated methods for characterizing cells using microscopic images have been developed, requiring the use of software such as CellProfiler^6^, ImageJ^7^, ImageJ macro Cell Colony Edge^8^, and OpenCFU^8^. In these methods, the images are treated by developer-defined procedures. Methods using machine learning to characterize cells in microscopic images have also been reported. For example, Flaccavento, G^9^ used random forest method for cell counting. Recently, a method using deep learning has also been reported. Thus, Khan, A^10^. directly counted the number of cells in a microscopic image after pretreatment using CNN. On the other hand, Xie, W^11^. and Cohen, JP^12^. used CNN to make a spatial density map from an image, and indirectly counted the cell number using the map. CNN technology has been incorporated in the characterization of cells using microscopic image data^13,14^.

However, a system to directly and accurately count the number of cells per well by inputting raw microscopic images with different boundaries of wells without any pre-treatment has not yet been established.

Since the accurate, high-throughput, automated counting of large numbers of cell samples is an inevitable technological development for single-cell experiments, here we report the initial development of such a method, fulfilling researcher requests.

## Results

The number of cells in 103 019 samples was determined visually by an independent double-check by two trained technicians. Assuming that the number of cells in a well follows a Poisson’s distribution, the parameter lambda was estimated by the maximum likelihood method to be 0.202 247. Proportions of wells with 0, 1, 2, and more than 2 cells were compared between technicians, and the values from the Poisson distribution with the lambda value of 0.202 247 (Supplementary Table 1). The proportion of the wells with single cells, as determined by the technicians, was slightly lower than that expected from the Poisson distribution. Conversely, the proportions of the wells with 2 or more than 2 cells were higher than when the same Poisson distribution was assumed (Supplementary Table 1). These results may suggest that the cells have divided or tend to adhere with each other to make clusters.

A single plate used for the experiment had 3984 wells, and we defined the data from a plate (3984 samples) as a subset of the entire samples. Among 30 subsets of the samples (119 520 total), we used 24 subsets for the training, 4 subsets for the validation, and 2 subsets for the test.

A mini-batch of 119 samples used to train the network was composed of 77, 21, 10, and 11 samples from categories corresponding to 0, 1, 2, and more than 2 cells, respectively. This partition was critical and a slight deviation from this partition resulted in a deviation from the correct result. For the training of the network, a mini-batch was sampled at random from the subsets used for the training. We defined a training process using a mini-batch an iteration.

**Figure 1:**
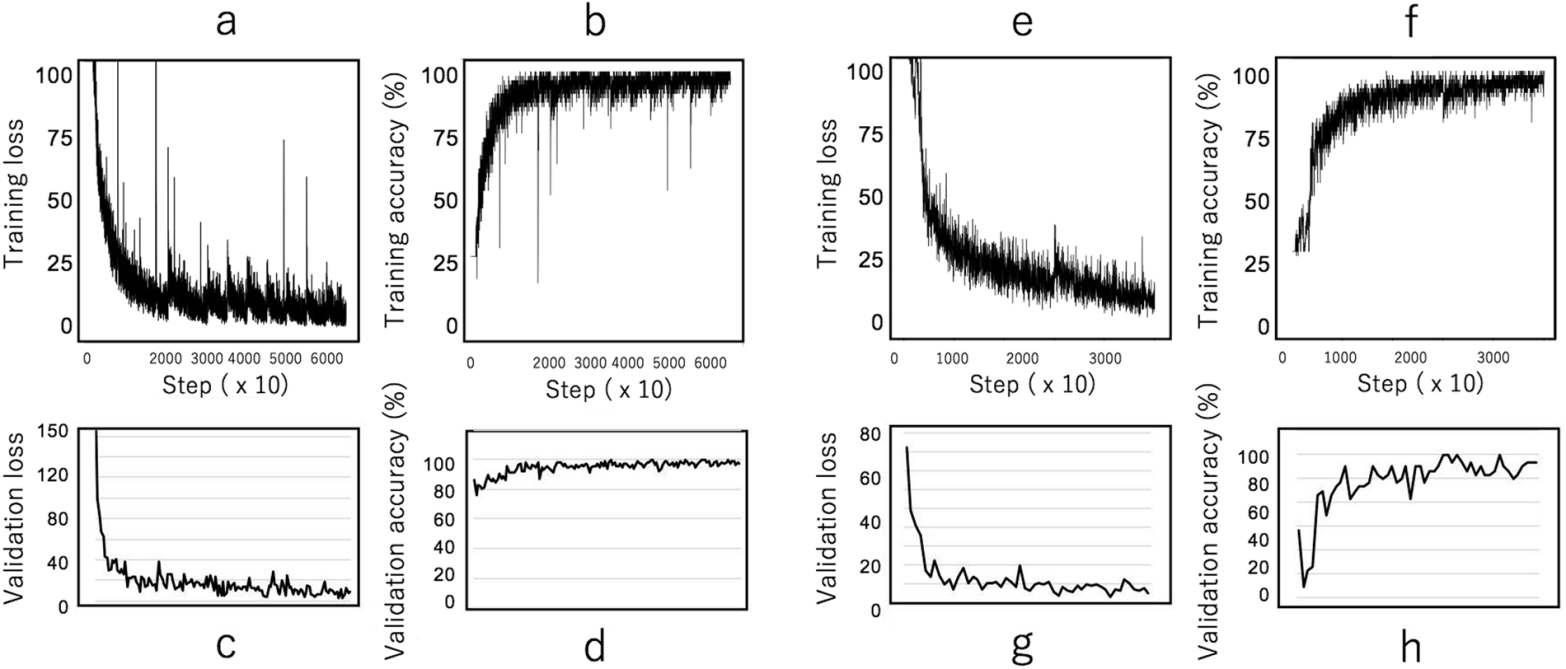
Stepwise change in loss and accuracy in the training process. Loss denotes the value of cross entropy at each step. The process of training with images of square-shaped wells is assessed by reference to changes in training loss (**a**), training accuracy (**b**), validation loss (**c**), and validation accuracy (**d**) with each iteration step. The process of training with images of round-shaped wells is assessed by reference to changes in training loss (**e**), training accuracy (**f**), validation loss (**g**), and validation accuracy (**h**) with each iteration step.

The first training of the network was performed for 20 000 iterations using the first 5 subsets (19 920 samples), followed by the second training for 10 000 iterations with the next 5 subsets. The third training was performed for 5000 iterations with the next 5 subsets. Thereafter, successive training processes were performed for 5000 iterations by randomly selecting 6 subsets from all of the 24 training subsets. The trained network was verified with the validation subsets, and learning was stopped before overfitting was observed (early stopping). We did not analyze all the samples simultaneously because of memory restriction. The time taken for an iteration was about 2.72 seconds. The training loss quickly decreased (Fig. 1a), training accuracy increased (Fig. 1b), validation loss decreased (Fig. 1c), and validation accuracy increased (Fig. 1d).

When independent data were used for the test, the overall accuracy of the prediction using the trained CNN was 98.3%. Table 1 shows the numbers and proportions of accurately classified nanoliter wells for different correct categories (according to trained technicians). The data indicate that the larger the number of cells, the smaller the correct prediction rate. Table 2 shows the numbers and proportions of the accurately classified nanoliter wells for different predicted categories. These data indicate that when the predicted number of cells are 0, 1, and 2, calculations are considered correct, having probabilities of 99.3, 95.6, and 85.8%, respectively. The proportions in Table 1 correspond to sensitivities and those in Table 2 correspond to positive predicted values.

**Table 1:** Proportions of accurately classified samples for different cell numbers determined by technicians.

Correct number of cells per well	Overall	0	1	2	>2
Number of wells according to trained technicians	7,920	6,458	1,225	209	28
Number of concordant wells*	7,787	6,420	1,150	193	24
Proportion	0.98321	0.99412	0.93878	0.92344	0.85714

^*^Number of wells in which answers from trained technicians and trained CNN were the same.

**Table 2:** Proportions of accurately classified samples for different predicted cell numbers by trained CNN.

Predicted number of cells per well	Overall	0	1	2	>2
Number of wells according to trained CNN	7,920	6,467	1,203	225	25
Number of concordant wells	7,787	6,420	1,150	193	24
Proportion	0.98321	0.99273	0.95594	0.85778	0.96

It takes approximately 40 min by technicians to visually determine the numbers of the cells in each well of one set 3984-well plate. In contrast, using the present CNN, results were obtained within 30 seconds, thereby indicating that our system is 80-fold faster than the visual method.

**Figure 2:**
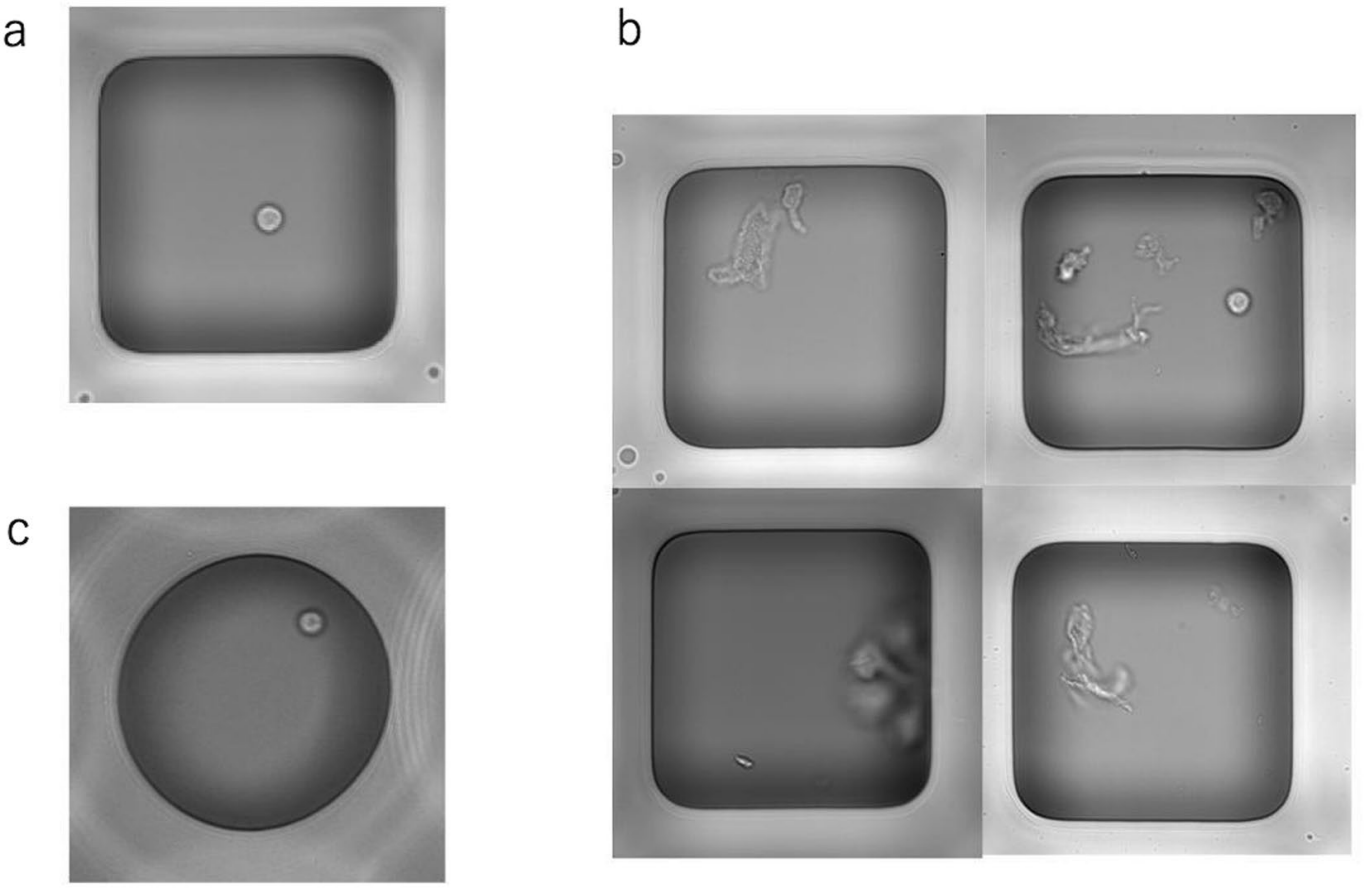
Images of nanoliter wells. (**a**) An example of a grayscale, 511 × 511 pixel digital image used as a sample. In this example, one cell is present in the well. (**b**) Examples of discordant well images. (**c**) An example 511 × 511-pixel image for the circular shaped nanoliter wells.

As shown in Tables 1 and 2, some of the well sample results were different between the trained CNN and the trained technicians, known as discordant wells or samples. Table 3 shows the discordance matrix that represents the number of discordant samples for different category combinations. Figure 2b shows examples of the images of the discordant wells. As shown in the figure, there are wells in which the cell counting is difficult due to the presence of debris or because objects are not well focused. We asked 10 people different from the trained technicians to visually determine the cell numbers of the discordant wells (133 wells) by observing images. Table 4 shows the concordance rate between the decisions by two technicians and the majority answers of the 10 people, and that between the decisions by trained CNN and the majority answers of the 10 people. Certainly the concordance rate when compared with the majority answers by 10 people was higher for decisions by two technicians than for trained CNN (p < 0.0001). Nonetheless, the concordance rates were lower than 0.7, thereby indicating that the discordant wells often have images whose cell numbers are difficult to determine.

**Table 3:** Discordance matrix showing the numbers of samples (wells) for which answers from technicians and CNN were different.

		Number of cells by trained CNN				
		0	1	2	over 2	Total
Number of cells by trained technicians	0	—	38*	0	0	38
	1	47	—	28	0	75
	2	0	15	—	1	16
	over 2	0	0	4	—	4
	total	47	53	32	1	133

^*^Number of wells for which decisions by trained technicians and CNN were different (discordant wells).

**Table 4:** Concordance rate between the majority answers by 10 people and decisions by two technicians or those by trained CNN.

Majority of 10 people vs	Two technicians	Trained CNN	p value*
Number of concordant wells	87**	36***	
Number of discordant wells	46	97	
Total	133	133	
Concordance rate (%)	0.65	0.27	<0.0001

^*^Chi square test.

^**^Number of wells in which the decisions were concordant between the majority answers by 10 people and two technicians.

^***^Number of wells in which the decisions were concordant between the majority answers by 10 people and trained CNN.

### Enhanced system

**Figure 3:**
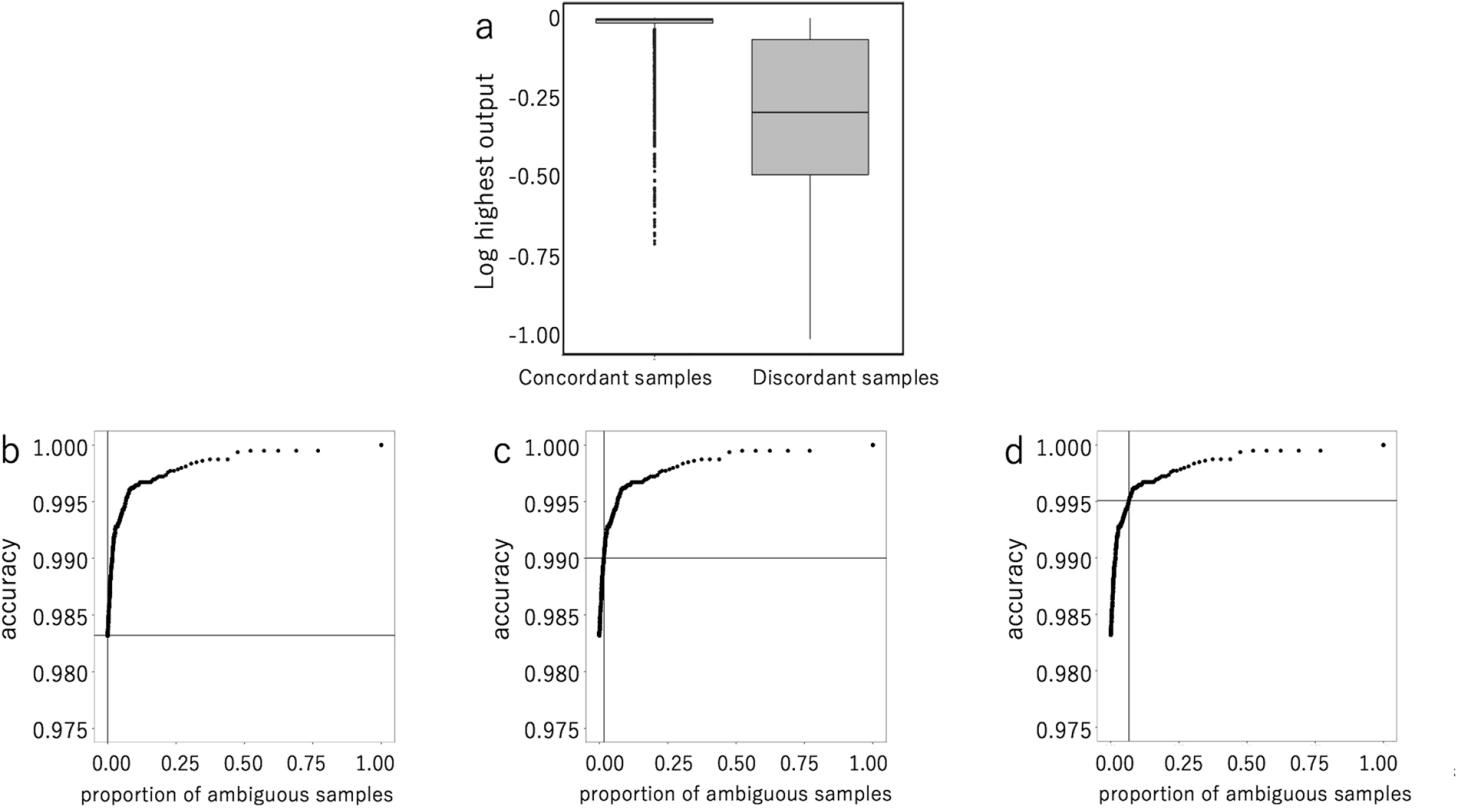
Defining ambiguous samples increases prediction accuracy. Ambiguous samples were defined by their highest outputs being lower than a threshold, and subsequently assessed by trained technicians. (**a**) A box plot comparison of log transformed highest outputs of concordant (7835) and discordant (133) samples. The results clearly indicate that discordant samples had much lower highest outputs as compared with concordant samples (P < 2 × 10^−16^, Mann-Whitney’s U test). (**b**–**d**) Change in the concordance rate between the results from the trained CNN and the trained technicians by changing the threshold value for the highest output from CNN for each sample. The samples with the highest outputs lower than the threshold value were classified as ambiguous samples. In this analysis, the ambiguous samples are assumed to be subsequently judged by technicians and all will become concordant. When the proportions of the ambiguous samples are 0 (**b**), 1 (**c**), and 5% (**d**), the concordance rates will become 98.3%, 99.0%, and 99.5% respectively.

Table 4 indicates that visual inspection may be superior to the trained CNN for some wells. The data suggest that the accuracy may be further improved by identifying wells with higher probabilities of discordance and subsequently assessing them visually. In the softmax regression, the final outputs are the posterior probabilities of different categories. Therefore, a sample with a higher discordance probability may be identified by evaluating the value of the highest output among categories. Figure 3a shows a comparison of log-transformed highest outputs of concordant and discordant samples. The results clearly indicate that discordant samples had much lower highest outputs than compared with concordant samples (P < 2 × 10^−16^, Mann-Whitney’s U test). By setting a threshold value for the highest output, the set of ambiguous samples was defined as {*s*_*i*_ | *h*(*s*_*i*_) < *t*} where *s*_*i*_ denotes the *i*th sample, *h*(*s*_*i*_) denotes the highest output for *s*_*i*_ and *t* denotes the threshold. Thus, we defined a set of samples whose highest outputs were lower than the threshold value in addition to the four categories concerning cell numbers. If the proportion of the ambiguous samples among the total number samples is not so large, only ambiguous samples require examination by visual inspection.

Figure 3b–d depict the changes in the concordance rates between the decisions made by CNN or trained technicians for different threshold values. In this analysis, the ambiguous samples are assumed to be subsequently judged by technicians and all will become concordant. The graph clearly shows that the higher the threshold value, the higher the proportion of ambiguous samples, and the higher the concordance rate. When the proportions of the ambiguous samples are 0 (Fig. 3b), 1 (Fig. 3c), and 5% (Fig. 3d), the concordance rates will become 98.3%, 99.0%, and 99.5%, respectively.

We further calculated the concordance rates for the samples when the ambiguous samples are excluded. The concordance rates thus calculated were also 98.3%, 99.0%, and 99.5% when the proportions of the ambiguous samples are 0, 1, and 5%, respectively.

For the routine use, the threshold can be a fixed value or can be changed according to the test samples. In the latter case, the threshold can be changed so that the proportion of ambiguous samples is 1%, 5%, 10% or any value. Although the accuracy becomes higher when the proportion of the ambiguous sample is increased, there is a trade-off between the accuracy and a load on trained technicians. Therefore, the appropriate threshold may be determined by considering the load on the technicians and the accuracy.

### Application of trained CNN to other well types

A different type of nanoliter well was used, having a circular shape with a 511 × 511 pixels image (Fig. 2c). Our CNN with the same network was trained by the image data from circular wells. The results showed that the training loss quickly decreased, the training accuracy increased, the validation loss decreased, and the validation accuracy increased (Fig. 1e–h). When independent data were used to assess accuracy, the overall accuracy (the proportion of concordant samples between technicians and CNN) of the trained CNN was 97.1%, without an extensive learning process. These results suggest that the presented CNN has high versatility and can be applied to the study of cell counting, irrespective of the well shape.

### Analysis by other machine learning algorithms

Next, the accuracy of the trained CNN was compared with that of other machine learning technologies, such as random forest and support vector machine learning. When independent data were used for the test, the support vector machine and random forest prediction accuracy were 75.1% and 77.2%, respectively (Supplementary Table 2). We used some different parameters for the above machine learning algorithms; however, extensive optimization of the parameters was not done. Although the optimization may not be sufficient, random forest and support vector machine learning were significantly inferior to the trained CNN in terms of accuracy.

In addition, we used multilayer perceptron, one of the deep learning methods, but the multilayer perceptron did not reach the level of CNN concerning the accuracy.

## Discussion

Although deep learning technology has been applied to microscopic cellular images, it has not been applied to count cell numbers in nanoliter wells for routine cellular experiments. For these experiments, a well should ideally contain a single cell, and cells are distributed in many wells so that the proportion of the wells with single cells is maximized. As a result, each well contains no cell, a single cell or a little more than a single cell. Therefore, the purpose of our CNN is different from previous studies^9–14^ although cell counting was performed in all of them. By applying a CNN to microscopic images of viable cells distributed into nanoliter wells, we have successfully constructed a system that can count the number of cells per well. The present automated system was trained by 103 019 samples and tested by independent 7968 samples. The results indicated that our trained CNN was much faster than visual inspection by two technicians, with comparable accuracies.

The system was further enhanced by using outputs from CNN, the posterior probabilities of the categories. Since the highest outputs among four categories were significantly higher for concordant samples than for discordant samples, the ambiguous category was added to the four categories in which the highest outputs were lower than the threshold value. A small proportion of the samples that were classified into the ambiguous category were either subsequently visually analyzed by trained technicians or excluded from further experiments. Our enhanced system had an accuracy over 99%. The researchers may check the histogram of log highest output from CNN and may decide the threshold so that the proportion (or the number) of ambiguous samples is not so large.

A limitation of the study is that the present system is assumed to be used in single-cell experiments, and identification of wells with clusters of cells or more than 3 cells is not supposed. Therefore, our system may not work as efficiently for crowded wells or wells with cell clusters as for the type of images used in our study. Furthermore, since only human ILC2 cells were used, the accuracy of the classification for other types of cells may not be as high as for human ILC2 cells. However, the prediction accuracy will be improved by training the CNN by using other cell types. Because of the high accuracies presented in this manuscript, we believe that the present algorithm will be useful for the classification of wells into different cell numbers, despite the assessment of other types of cells.

In this study, we used a special iteration technique as described in the method due to our low memory. By using this method, researchers with equivalent computational capacity will be able to archive the research using equivalent large-scale input images.

In the future, the present method may be useful for various large-scale cellular experiments in high-throughput or high-content screening.

In conclusion, we constructed a system, for single-cell experiments, using a deep learning algorithm to determine the number of cells per well directly from the raw microscopic well image data without pre-treatments as accurately as the visual method, but within a much shorter time. The system was further enhanced by defining the ambiguous samples to be subsequently examined by visual inspection.

## Methods

### Data

The data used were obtained from experiments involving the real-time single-cell imaging of protein secretion developed by Shirasaki *et al*.^15^. This method is a new assay platform that can monitor real-time protein secretion from single cells. In the present study, type 2 innate lymphoid cells (ILC2s) were used as a cell type suitable for single-cell imaging^16^. ILC2s are a subset of natural lymphocytes that have attracted the attention of researchers in recent years. Reportedly, the ILC2 numbers in sputum and blood of severe asthmatics are elevated compared with those in mild asthmatics, suggesting that ILC2s contribute to the severe asthma phenotype^17^.

Approximately 100s–5000 ILC2s were collected from 20 ml of human peripheral blood, and the collected cells were distributed into nanoliter wells so that only one cell was present in each well, suited to functional analysis at the single-cell level. Approximately 700 cells were uniformly distributed into a plate with 996 × 4 nanoliter wells. Thereafter, the researchers added cytokines (IL-2/IL-25, IL-2/IL-25/TSLP, IL-2/IL-33/TSLP, IL-2/TSLP) to the wells and observed changes in cell proliferation and morphology. Using this platform, the secretion of cytokines can be measured as a time series; however, since this process is not relevant the present study, details are not described in the present manuscript. As shown in Fig. 1a, the digital image of each nanoliter well is obtained as a gray scale image with 511 × 511 pixels.

It is not difficult to determine the number of cells in a well by visual inspection (Fig. 2a). However, the number of nanoliter wells in each experiment is 3984 × the number of time-series measurements, making it necessary to visually determine the number of cells in a large number of wells for a single experiment. Because of the huge number of nanoliter wells per experiment, counting the number of cells by visual inspection is cumbersome and error-prone. Therefore, methods are required to automatically count the number of cells in a well accurately.

### Algorithm Development

Deep learning is a multilayered neural network. The process of deep learning is divided into two parts, i.e., unsupervised learning that does not need correct answers and supervised learning that needs correct answers. For the latter, parameters are optimized so that the outputs are as close to the correct answers as possible. In our study, however, unsupervised learning was not implemented because CNNs do not usually require it. Deep learning is often applied to a huge number of samples with multidimensional quantitative data that are difficult to separate with linear algebra. Deep learning uses a complicated non-linear function with an enormous number of parameters that should be optimized so that the outputs predict the given supervisor’s answers as accurately as possible.

The function used in our study outputs the posterior probabilities (softmax functions) of the below mentioned four categories, each corresponding to a number of cells in a nanoliter well, by inputting the grayscale level of 511 × 511 pixels each with 8-bit data as the microscopic image. The softmax regression method was used to optimize parameters based on a large number of samples. Regression was performed using the gradient descent algorithm based on the backpropagation method that updates the parameters in each iteration (trained by the training data) so that loss function is minimized. Loss denotes the value of cross entropy.

The initial neural network parameters were sampled from a normal distribution with a mean of 0 and a variance of 2/Nin (Nin denotes the number of nodes of the previous layer in the network). This method has been reported to show good learning accuracy^18^ and is generally used for CNNs.

Apart from the input and output layers, the CNN was composed of 18 layers, namely 8 convolution layers, 4 pooling layers, 4 local response normalization (lrn) layers, and 2 totally connected layers (Table 5). A rectified linear unit was used as the activation function for both convolution layers and totally connected layers (except for the output layer). Dropouts were used for each totally connected layer for preventing overfitting by randomly setting 30% of all nodes to zero in the training step. The total number of parameters was 468 768 (Table 5).

**Table 5:** Architecture of the network.

Layer description	No of nodes	No of Weights
Input	511 × 511	
Local respose normalization 1	511 × 511	
Max pooling 1	256 × 256	
Convolution 1	32 × 256 × 256	5 × 5 × 1 × 32
Convolution 2	32 × 256 × 256	5 × 5 × 32 × 32
Max pooling 2	32 × 128 × 128	
Local respose normalization 2	32 × 128 × 128	
Convolution 3	64 × 64 × 64	5 × 5 × 32 × 64
Convolution 4	64 × 64 × 64	5 × 5 × 64 × 64
Convolution 5	64 × 64 × 64	5 × 5 × 64 × 64
Max pooling 3	64 × 32 × 32	
Local respose normalization 3	64 × 32 × 32	
Convolution 6	32 × 16 × 16	5 × 5 × 64 × 32
Convolution 7	32 × 16 × 16	5 × 5 × 32 × 32
Convolution 8	32 × 16 × 16	5 × 5 × 32 × 32
Max pooling 4	32 × 8 × 8	
Local respose normalization 4	32 × 8 × 8	
Totally connected 1	32	32 × 32 × 8 × 8
Totally connected 2	512	512 × 32
Output	4	4 × 512
Total		468,768

For the computation, NC 6 (NC series, 6 core, RAM 56.00 GiB, storage 340 GB, GPU 1 × NVIDIA Tesla K 80) of the Ubuntu Server 16.04 LTS was used through Virtual machines of Microsoft Azure. Python (version 3.5.2) and the TensorFlow library (version 0.12.0rc1) were also used.

The training method was as follows. The input layer was composed of 261 121 (511 × 511) nodes showing the density of pixels in an image. The output layer was composed of 4 nodes corresponding to categories of 0, 1, 2, and more than 2 cells per well. Since the wells with four or more cells were extremely rare, it was considered to be appropriate to define a category for more than 2 cells per well. The accuracy of the trained system classification was judged by using a set of test samples independent of the training set. The images originally derived as jpeg2000 files were converted to text files and then normalized such that the minimum was 0 and the maximum was 255, to adjust for a slight deviation in values due to differences in conditions such as the intensity of light. Furthermore, in order to augment the input data, images were randomly inverted or rotated by 90, 180, or 270 degrees in about 20% of the cases.

Since the morphology of the cells changed with time, and image data were obtained at different times, the image data were selected at random from those images obtained at different times.

### Algorithm Evaluation

The trained CNN outputted the posterior probabilities that the cell numbers are 0, 1, 2, and more than 2, respectively, and the category with the highest value was interpreted as the category predicted by the CNN. The accuracy was calculated as the number of concordant samples divided by the total number of inputted images. A concordant sample means that the answers obtained for that sample from two trained technicians and our trained CNN are the same. In addition, accuracy was also examined for each correct cell number and for each predicted cell number in order to identify possible biases between the categories.

## Electronic supplementary material

supplementary table
