# Supplementary material for: Construction of a system using a deep learning algorithm to count cell numbers in nanoliter wells for viable single-cell experiments
Source: Sci Rep. 2017 Dec 4;7:16831. doi: 10.1038/s41598-017-17012-x (PMC5715092; doi:10.1038/s41598-017-17012-x)
Supplement: Supplementary file 1 — supplementary table [file 41598_2017_17012_MOESM1_ESM.pdf]

Construction of a system using a deep learning algorithm to count cell numbers in nanoliter wells for viable single-cell experiments

Takashi Kamatani, Koichi Fukunaga, Kaede Miyata, Yoshitaka Shirasaki, Junji Tanaka, Rie Baba, Masako Matsusaka, Naoyuki Kamatani, Kazuyo Moro, Tomoko Betsuyaku, Sotaro Uemura

Supplementary Table 1 Comparison of the proportions of wells with different cell numbers between the decisions by two technicians and the Poisson distribution

No of cells/well	0	1	2	More than 2	Total
No of wells	84,193	16,007	2,367	452	103,019
Proportion*	0.81726	0.15538	0.02298	0.00439	1
Poisson**	0.81689	0.16521	0.01671	0.00119	1
Expected number***	84,155	17,020	1,721	123	103,019

*Proportion according to the decisions by technicians

**Proportion expected from Poisson distribution with lambda = 0.202247

***Expected number of wells when Poisson distribution was assumed.

Supplementary Table 2 Comparison of accuracy between different multiple machine learning algorithms

Machine learning system	Accuracy for test data
Our trained CNN	0.98321
Support vector machine	0.75063
Random forest	0.77206
